# Trajectories and predictors of social avoidance in female patients with breast cancer

**DOI:** 10.3389/fpsyt.2022.1051737

**Published:** 2022-11-25

**Authors:** Chunyan He, Chao Wu, Tianqi Yang, Yang He, Jiaran Yan, Yawei Lin, Yanling Du, Shizhe He, Shengjun Wu, Baohua Cao

**Affiliations:** ^1^Department of Nursing, Fourth Military Medical University, Xi’an, Shaanxi, China; ^2^Department of Psychology, Fourth Military Medical University, Xi’an, Shaanxi, China

**Keywords:** social avoidance, breast cancer, trajectory, growth mixed model, nursing

## Abstract

**Background:**

Social avoidance plays an important role in influencing quality of life among patients with breast cancer. Social avoidance behaviors change with treatment periods. However, the trajectory patterns and the predictive factors have not been fully studied.

**Objective:**

This study examined the growth trajectory of social avoidance and its predictors in patients with breast cancer.

**Materials and methods:**

A total of 176 patients with breast cancer in a university hospital in Shaanxi Province, China, were followed up four times over 6 months following surgery, and data from the final 144 patients were analyzed. The growth mixed model (GMM) was used to identify the trajectory categories, and the predictive factors of the trajectory types were analyzed by logistic regression.

**Results:**

The best-fit growth mixture modeling revealed three class models: persistent high social avoidance group (Class 1), social avoidance increased first and then decreased group (Class 2), and no social avoidance group (Class 3), accounting for 13.89, 31.94, and 54.17% of patients, respectively. Single-factor analysis showed that family income per capita, residence, and temperament type were related to the social avoidance trajectory. Logistic regression analysis showed that only temperament type was an independent predictor of the social avoidance trajectory, and patients with melancholia were more likely to have persistent high social avoidance.

**Conclusion:**

Our study proved the heterogeneity of social avoidance behaviors and the influencing effect of temperament type on the development of social avoidance behaviors in Chinese patients with breast cancer. Health professionals should pay more attention to patients who are at higher risk of developing a persistent social avoidance pattern and provide target interventions.

## Introduction

Breast cancer is the most common cancer among women worldwide (1). With the improvement of early diagnosis and treatment, the 5-year relative survival rate of breast cancer patients has been increasing continuously, up to 90% (2). Longer survival times put higher demands on patients’ physiological and psychological considerations both during and after treatment (3), and consolidation of treatment effects and improvement of posttreatment well-being have become urgent issues in breast cancer patients. However, the treatment of breast cancer surgery creates psychological distress, such as negative emotions, distorted self-image, repressed sexual behavior, and the generation of low self-esteem (4). In addition, mastectomy and the loss of femininity make patients feel unconfident and discriminated against (5), and they often adopt negative coping styles in social interactions, leading to the emergence of social avoidance problems (6–8). Therefore, as an irrefutable fact, breast cancer patients have a high risk of social avoidance (7).

Social avoidance is defined as the avoidance of interaction, conversation, or contact with another person for any reason, including actual avoidance behavior and avoidance tendencies (9). Social avoidance is a serious threat to the mental health of patients and a burden to society (10). In addition, patients with breast cancer, especially young patients with breast cancer (11), pay more attention to appearance changes, after the acute survival period (the period of cancer diagnosis and treatment); later, the whole survival period will present a series of social problems, such as career choice, marriage, and social interaction (12). However, due to existing or potential health problems, patients with breast cancer are often in a weak or passive position in social interactions (13). According to DSM-5 (14), as one of the core symptoms in schizoid personality disorder and avoidant personality disorder, social avoidance has been shown to predict the transition to psychosis and usually occurs before other symptoms (15, 16). However, researchers have mainly focused on pathology, ignoring the social avoidance caused by disease (17). In addition, translational research on the social avoidance of human beings has been largely ignored due to difficulties in scheduling ecologically valid social threats in the laboratory (18). Therefore, paying attention to the social avoidance problems of patients with breast cancer is of significance for improving their quality of life and helping them reintegrate into society.

Social avoidance in patients with breast cancer seriously affects their psychological experience and quality of life. Furthermore, research has shown that social avoidance by breast cancer patients is in the process of development (19). For most patients, breast cancer diagnosis is a traumatic event and usually induces symptoms of stress disorders such as avoidance or fear at the beginning (19, 20). Individuals exposed to traumatic events may experience posttraumatic stress (PTS) and posttraumatic growth (PTG) over time (21). PTS symptoms consist of intrusive thoughts, hypervigilance, and mood changes, and persistent symptoms may eventually lead to posttraumatic stress disorder (PTSD) (22) and represents function adjustment beyond the prevent level (23). Therefore, PTS may lead to or aggravate social avoidance behaviors as a manifestation of stress disorder in breast cancer patients over time. PTG refers to positive psychological and behavioral changes that occur after a traumatic event (24). PTG may prevent or reduce social avoidance behavior in patients with breast cancer over time. Although seemingly incompatible, PTS and PTG often occur simultaneously in patients with breast cancer (25). However, the occurrence of PTS and PTG after trauma may be influenced by many factors, such as sociodemographic factors, disease-related factors, and personality traits (26). Therefore, based on the development theory of PTS and PTG, we hypothesized that social avoidance behavior in different patients with breast cancer may develop different trajectories over time.

As a significant impact on the development of posttraumatic psychopathology (27), personality is a set of traits and styles that reflect persistent differences between an individual and the “standard normal person” in the social environment (28). As one of the remarkable achievements of personality research, the Eysenck personality model constructs a pyramid structure to describe the construction of personality associated with specific reactions, habitual reactions, traits, and dimensions (29). In this model, personality is a combination of three dimensions: neuroticism, extroversion, and psychoticism. Previous studies related to personality and psychopathology have shown that high extraversion significantly predicts high PTG, while low neuroticism significantly predicts low PTS (30, 31). Personality traits also provide a basis for different posttraumatic appearances of breast cancer patients through the diathesis-stress model. Patients with high neuroticism pay too much attention to their own feelings and are prone to depression and avoidance in the face of difficulty, while patients with high extroversion tend to achieve PTG and bravely face social interactions. As the core of personality, temperament is relatively stable and can predict psychological problems that have not occurred yet. Hassan and Schmidt (32) proposed the possibility that temperament types can predict trajectories of avoidance in preschoolers. Can temperament types influence the development trajectory of social avoidance in patients with breast cancer? We have explored this in the present study.

Previous research on social avoidance among patients with breast cancer has two major shortcomings. First, most of the studies were mainly cross-sectional, and such research methods can hardly reflect the trajectory of changes in social avoidance in patients with breast cancer. Second, previous studies on posttraumatic changes have tended to treat patients’ psychological states as uniformly distributed, ignoring the issue of individual differences (33, 34). Therefore, there is an urgent need for a new approach to address the shortcomings in previous studies, and the growth mixture model (GMM) offers a promising approach to address these deficiencies. First, GMM is a common statistical method that groups heterogeneous populations according to their development trajectories (35, 36); second, GMM can provide methods to identify which subset of a variable based on the trajectory distinction is more susceptible to the influence of another variable and to clarify the non-linear relationship between these variables (37); third, the superiority of GMM over other statistical methods has been confirmed by simulation studies, such as latent class growth analysis and latent class analysis (38–40). Given previous studies on the effects of interventions on social avoidance, increasing the understanding of social avoidance trajectories and providing targeted interventions are critical for early intervention and prognostic outcomes (41, 42). Therefore, we explored the developmental trajectory of social avoidance in patients with breast cancer based on GMM and analyzed the relationship among the developmental trajectory of social avoidance, sociodemographic factors, disease-related factors, and temperament type. Finally, our study can provide a theoretical reference for targeted interventions for social avoidance in patients with breast cancer and has important implications for the physical and psychological rehabilitation of breast cancer patients.

## Materials and methods

### Participants

In this study, convenience sampling was used to choose breast cancer patients who had undergone surgery in a grade III, Class A hospital in Xi’an, Shaanxi Province, from June 2021 to April 2022. The inclusion criteria were as follows: (a) female inpatients; (b) aged 18–60 years old; (c) diagnosed with breast cancer by pathological puncture biopsy and scheduled for surgery; and (d) conscious, without cognitive dysfunction, psychiatric disorders or communication disorder, who provided their informed consent and volunteered to participate in the study. The sample was excluded twice in this study, once at the initial survey, using the baseline exclusion criteria, and a second time during follow-up, using the follow-up exclusion criteria. The baseline exclusion criteria were as follows: (a) recurrence of breast cancer during treatment; (b) other serious concomitant diseases, such as other cancers and cardiopulmonary insufficiency; and (c) distant metastasis that had occurred at the time of diagnosis. The follow-up exclusion criteria were as follows: (a) metastasis occurring during follow-up; and (b) failure to follow-up *via* telephone on three consecutive occasions. The research followed the ethical principles of the Declaration of Helsinki, and ethical approval (KY20192117-F-1) was obtained by the Ethics Committee of the First Affiliated Hospital of the Fourth Military Medical University. All patients signed informed consent forms.

This prospective study investigated eligible breast cancer patients at four time points. The calculation of the sample size refers to the table used for sample size estimation in the design of single-group repeated measurements (43). Four measurements per patient were selected with a mean correlation coefficient of *r* = 0.5, *f* = 0.14 (weak effect), α = 0.05, and a sample size of 142 patients was required under conditions that ensured 1 − β = 0.8.

### Measures

#### The demographic and clinical questionnaire

The researchers designed the demographic and clinical questionnaire and included two parts: sociodemographic and clinical information. It included age, educational level, occupation, marital status, family monthly income per capita, radiotherapy and chemotherapy, operation method and temperament type.

#### The Chinese version of the Eysenck personality questionnaire short form

The questionnaire was developed by Eysenck and Eysenck (44) and introduced in China in 2000 by Qian et al. (45); it mainly includes the neuroticism subscale, extraversion subscale, lie detection subscale and psychoticism subscale, each with 12 items for a total of 48 items. In this study, temperament types were classified according to neuroticism subscale and extroversion subscale. The scoring of each subscale was scored with “Yes” (0) or “No” (1), and the subscale ranged from 0 to 12. Lower total scores in each category indicated a higher degree of extraversion and neuroticism. The main method was to take the outward type as the transverse axis and neuroticism as the longitudinal axis, which then forms four quadrants. According to the scores, the patients were divided into four major temperament types: high extraversion and low neuroticism (sanguineous), high extraversion and high neuroticism (choleric), low extraversion and low neuroticism (lymphatic), and low extraversion and high neuroticism (melancholic). The Cronbach’s α coefficients of the subscales used in this study were 0.75 and 0.77 (45), respectively. It was proven that the scale had good reliability.

#### The Chinese version of social avoidance and distress scale

The scale was developed by Watson and Friend (46) and introduced in China in 1999 by Wang et al. (47), including a social avoidance subscale and a social distress subscale. In this study, social avoidance was assessed according to the social avoidance subscale. For the social avoidance subscale (including 14 items), patients responded with “Yes” (0) or “No” (1), and the subscale ranged from 0 to 14. A score of ≥ 7 indicates social avoidance; the higher the score is, the more likely the patients are to avoid social interactions. The Cronbach’s α coefficient of this subscale is 0.834 (48), indicating good reliability.

### Procedure

This prospective study investigated eligible breast cancer patients at four time points: before surgery (wave 1), 1 month after surgery (wave 2), 3 months after surgery (wave 3), and 6 months after surgery (wave 4). The researchers first conducted a semistructured interview with patients who met the inclusion criteria, established a good relationship, and informed them of the purpose of the study and follow-up arrangements. After the patients provided informed consent, the researchers collected the baseline data. After discharge, patients were followed up regularly according to plan. At the same time, to ensure the accuracy and reliability of the data, follow-up appointments were fixed within 1 week during the follow-up period, and the follow-up timepoints were 9:00–11:00 or 16:00–18:00. A total of 176 patients were included in this study. Of the 176 eligible patients recruited, all gave informed consent, and ultimately 144 (response rate: 81.82%) patients completed all follow-ups, 5 (2.84%) had a postoperative recurrence, 13 (7.39%) clearly expressed no interest in continuing the study, and 14 (7.95%) were lost to follow-up because of loss of contact.

### Statistical analyses

The data were analyzed using SPSS 25.0 and Mplus 8.3 software. We used the method of full information maximum likelihood to handle missing data. First, when the model was constructed, the unconditioned latent class growth model (LCGM) and GMM were used to judge the trajectory categories with Mplus 8.3 software, and LCGM is a special form of GMM. The baseline model is a single-category model, setting the variance within the category to 0, increasing the number of categories in the model one by one, and then comparing the fitting indexes between the models. The optimal model is determined by combining the practical significance and statistical indices (49). Fit indicators include the akaike information criterion (AIC), bayesian information criterion (BIC), adjusted BIC (aBIC), entropy, likelihood ratio test (LRT), and bootstrapped likelihood ratio test (BLRT). The smaller the AIC, BIC, and aBIC values are, the better the model fit. Entropy represents the accuracy of classification, and the higher the entropy value is, the more accurate the classification. LRT and BLRT are commonly used to compare fit differences between K-1 and K-category models, with *P* < 0.05 indicating that the K-category model is superior to the K-1-category model. The category to which an individual belongs is then determined based on a posterior probability. Second, according to the fitting results of the individual category model, the above indexes were comprehensively evaluated, the best fitting model was selected, and the patients were divided into different categories. Then, SPSS 25.0 was used for analysis. Data that followed a normal distribution were expressed as the mean and standard deviation, and analysis of variance was used to compare multiple groups. Count data were expressed by frequency and constituent ratio, and the chi-square test was used to compare multiple groups. Finally, logistic regression analysis was used to explore the influence of social demography, disease-related data and temperament types on the class of social avoidance. There was a significant difference at *P* < 0.05.

## Results

### Descriptive statistics

A total of 144 cases of breast cancer were included in this study. All of them were female, aged 21–60 (42.67 ± 13.84), 93.75% were married, 36.11% had a junior high school education or less, 44.44% of the patients had a monthly income of less than 3000 yuan, 34.72% were unemployed, 31.94% of them lived in rural areas, 82.64% of them had experienced chemotherapy, 59.72% of them had experienced radiotherapy, 59.72% of the patients underwent a radical mastectomy, and 20.83% of the patients were choleric.

### Identification of the trajectories of social avoidance in patients with breast cancer

Taking the social avoidance score of patients with breast cancer at four time points as an observation index, data from 144 patients were included in the model analysis. First, LCGM was used and set as the free estimation of the time parameter, and 1∼5 categories were extracted in turn. When the number of potential classes increased from 1 to 5, AIC, BIC, and aBIC all decreased. However, when the number of categories increased from 3 to 4, entropy decreased, and LRT and BLRT became insignificant, suggesting that the best fit was to retain three categories. To further judge the optimal model, the model is set to GMM, the model of Category 2∼5 has good fit, AIC, BIC, aBIC values are less than the values of LCGM, suggesting that the model was optimized, but the entropy decreased, LRT and BLRT values were not significant when extracting 4 and 5 categories. Based on the above information, combined with the theoretical background of social avoidance trajectories, the classification probability of the model and the interpretability of the results, the three categories of LCGM were retained. The results of model fitting are shown in Table 1.

**TABLE 1:** The results of model fitting (*n* = 144).

Model	K	AIC	BIC	aBIC	Entropy	LRT	BLRT	Class percentage
						*P*	*P*	
LCGM	1	3161.943	3182.732	3160.582	–	–	–	1
	2	2893.887	2923.585	2891.943	0.909	< 0.001	< 0.001	43.06/56.94
	3	2825.681	2864.688	2823.153	0.921	< 0.001	< 0.001	54.17/13.89/31.94
	4	2779.883	2827.400	2776.772	0.880	0.0875	< 0.001	29.86/17.36/28.47/24.31
	5	2743.505	2799.931	2739.810	0.903	0.2373	< 0.001	26.39/4.86/25.00/14.58/29.17
GMM	2	2757.200	2795.808	2754.672	0.803	0.0352	< 0.001	37.50/62.50
	3	2735.302	2782.819	2732.191	0.849	0.0120	< 0.001	56.25/36.81/6.94
	4	2723.022	2779.448	2719.327	0.886	0.2526	< 0.001	31.25/10.42/52.08/6.25
	5	2723.353	2788.689	2719.075	0.875	0.5088	0.2552	47.92/27.08/5.56/3.47/15.97

AIC, akaike information criterion; BIC, bayesian information criterion; aBIC, sample size adjusted BIC; LRT, likelihood ratio test; BLRT, bootstrapped likelihood ratio test.

**FIGURE 1:**
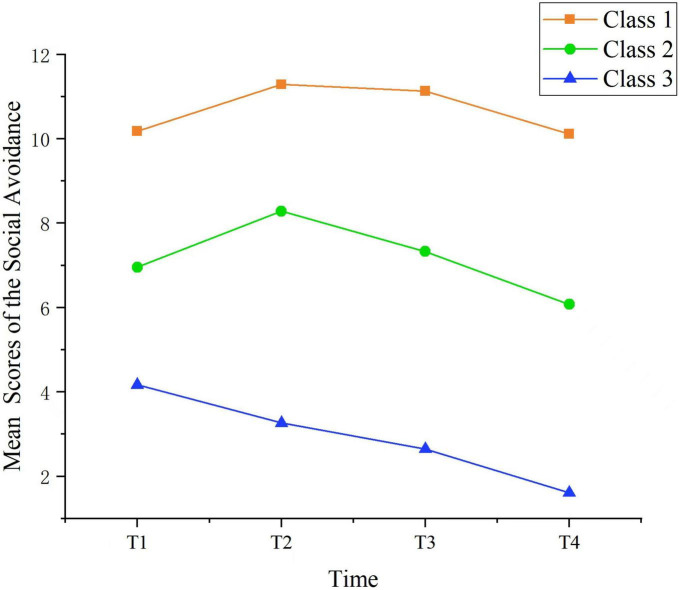
Three classes of growth trajectory of social avoidance.

Each class was named according to its social avoidance score and changing trend. Class 1 (13.89%) had consistently higher scores, but the mean change was not significant throughout the follow-up period (*P* = 0.163). Therefore, class 1 was named the “persistent high social avoidance group.” In Class 2 (31.94%), the scores increased and then decreased (*P* < 0.001), so this group was named “social avoidance increased first and then decreased group.” The score of Class 3 (54.17%) was the lowest, and all classes were below 7 points; the scores decreased continuously during follow-up (*P* < 0.001), and this group was named “no social avoidance group.” The three trajectories of social avoidance from the model fit are shown in Figure 1.

### Single factor analysis of influencing factors of social avoidance trajectories in patients with breast cancer

Single factor analysis was used to identify the possible predictive factors according to the different categories determined by LCGM, with *P* < 0.05 indicating a significant difference. The general data and temperament type were included in the single factor analysis. The results showed that the family monthly income per capita (RMB) (*X*^2^ = 10.545, *P* = 0.032), residence (*X*^2^ = 8.433, *P* = 0.015) and temperament type (*X*^2^ = 41.542, *P* < 0.001) were significantly different among the three groups. The mean ages of the three groups were 44.85 ± 7.343, 48.26 ± 8.296, 46.12 ± 8.688, and *F* = 1.262, respectively (*P* = 0.188), and there was no significant difference among the three groups. The difference between the other variables was not significant. The results are shown in Table 2.

**TABLE 2:** Single-factor analysis of influencing factors of social avoidance trajectories (*n* = 144).

Variables	Class 1	Class 2	Class 3	X^2^	*P*
**Marital status**					
Married	19 (0.95)	44 (95.65)	72 (92.31)	0.614	0.736
Unmarried/divorced/ widowed	1 (0.05)	2 (4.35)	6 (7.69)		
**Educational level**					
Junior secondary and less	11 (0.55)	17 (36.96)	24 (30.77)	6.338	0.175
High school/junior college	8 (0.40)	17 (36.96)	32 (41.03)		
Bachelor and more	1 (0.05)	12 (26.08)	22 (28.20)		
**Family monthly income per capita (RMB)**					
<3000	14 (0.70)	20 (43.48)	30 (38.46)	10.545	0.032
3000∼5000	5 (0.25)	19 (41.30)	25 (32.05)		
>5000	1 (0.05)	7 (15.22)	23 (29.49)		
**Occupation**					
Enterprises/institutions	2 (0.10)	13 (28.26)	26 (33.33)	7.620	0.267
Laborer	5 (0.25)	8 (17.39)	13 (16.67)		
Retired	2 (0.10)	9 (19.57)	16 (20.51)		
Unemployed	11 (0.55)	16 (34.78)	23 (29.49)		
**Place of residence**					
Urban area	8 (0.40)	33 (71.74)	57 (73.08)	8.433	0.015
Rural area	12 (0.60)	13 (28.26)	21 (26.92)		
**Chemotherapy**					
Yes	18 (0.90)	35 (76.09)	66 (84.62)	2.344	0.310
No	2 (0.10)	11 (23.91)	12 (15.38)		
**Radiotherapy**					
Yes	14 (0.70)	28 (60.87)	44 (56.41)	1.259	0.533
No	6 (0.30)	18 (39.13)	34 (43.59)		
**Surgical method**					
Radical mastectomy	14 (0.70)	29 (63.04)	43 (55.13)	2.416	0.660
Breast-conserving	5 (0.25)	12 (26.09)	23 (29.49)		
Breast reconstruction	1 (0.05)	5 (10.87)	12 (15.38)		
**Temperament type**					
Choleric	4 (0.20)	12 (26.07)	14 (17.95)	41.542	<0.001
Sanguineous	3 (0.15)	14 (30.43)	52 (66.67)		
Melancholic	12 (0.60)	13 (28.26)	4 (5.13)		
Lymphatic	1 (0.05)	7 (15.22)	8 (10.26)		

### Logistic regression analysis of influencing factors of social avoidance trajectory categories in patients with breast cancer

The social avoidance trajectories of patients with breast cancer determined by LCGM were used as the dependent variable, and Class 3 was used as the control group. The independent variables were assigned as follows: family monthly income per capita (RMB): 0 = “<3000,” 1 = “3000∼5000,” 2 = “>5000”; place of residence: 0 = “urban area,” 1 = “rural area”; temperament type: 0 = “choleric,” 1 = “sanguineous,” 2 = “melancholic,” 3 = “lymphatic.” Logistic regression analysis of social avoidance trajectory categories in patients with breast cancer is shown in Table 3.

**TABLE 3:** Logistic regression analysis of social avoidance trajectory categories in patients with breast cancer (*n* = 144).

Variables	Class 1 vs. Class 3				Class 2 vs. Class 3			
	β	OR	95% CI	*P*	β	OR	95% CI	*P*
**Family monthly income per capita (RMB)**								
<3000	2.010	7.462	(0.722, 77.131)	0.092	0.751	2.119	(0.674, 6.659)	0.199
3000∼5000	1.664	5.278	(0.470, 59.205)	0.177	1.023	2.783	(0.888, 8.718)	0.079
>5000	–	1.000	–	–	–	1.000	–	–
**Place of residence**								
Urban area	−0.972	0.378	(0.109, 1.319)	0.127	0.121	1.128	(0.453, 2.808)	0.795
Rural area	–	1.000	–	–	–	1.000	–	–
**Temperament type**								
Choleric	0.957	2.603	(0.234, 28.960)	0.436	0.076	1.079	(0.295, 3.952)	0.908
Sanguineous	−0.571	0.565	(0.050, 6.372)	0.644	−1.137	0.321	(0.097, 1.062)	0.063
Melancholic	3.319	27.619	(2.425, 314.579)	0.008	1.384	3.992	(0.857, 18.594)	0.078
Lymphatic	–	1.000	–	–	–	1.000	–	–

## Discussion

### There were different trajectories of social avoidance in patients with breast cancer

This study identified three different trajectories of social avoidance in breast cancer patients based on the GMM, namely, Class 1: “Persistent high social avoidance group,” Class 2: “Social avoidance first increases and then decreases group,” and Class 3: “No social avoidance group,” accounting for 13.89, 31.94, and 54.17% of patients, respectively, proving the development theory of PTS and PTG.

The proportion of Class 1 was 13.89%, in which patients were in a state of high social avoidance for a long time. This is similar to previous longitudinal studies of negative emotions in breast cancer patients. A study such as Henselmans’s et al. (50) found that 15.2% of patients were in the group with persistently high levels of mental distress. Kant et al. (51) also found that 11.3% of patients were in the group with persistently high anxiety levels. This study also confirms Dorsett’s (52) recovery trajectory theory that negative emotions and negative behaviors persist in a small number of patients. Patients who persist in a negative state are more likely to adopt passive coping styles such as yield and avoidance (53), which may hinder them from returning to society. Moreover, a previous study (54) showed that patients with severe social avoidance have a significant decline in learning, social and work social skills, which has a serious impact on patients’ return to society and places a heavy burden on families and society. Therefore, these patients are also the clinical key observation object, and medical staff need to recognize and pay close attention to them. The proportion of Class 2 was 31.96%. This may be because some patients do not have impaired baseline bodily integrity and because physical disability increases feelings of inferiority and stigma after surgery progresses (55), which also leads to social withdrawal, social avoidance, and even social phobia (56). This is consistent with the findings of Xu et al. (7), who found that breast cancer patients experience severe social withdrawal and distress during the acute survival period, especially after mastectomy.

We found that approximately half of all breast cancer patients experience social avoidance, which suggests that breast cancer patients have a high risk of social avoidance. Therefore, medical staff should pay attention to the early identification of patients with social avoidance and provide group therapy using guided imagery and education to reshape their body image (57).

### Melancholic temperament can predict the trajectories of social avoidance in patients with breast cancer

This study found that compared to patients with other temperament types, patients with melancholic temperament had worse mental health and were more likely to have persistent social avoidance, confirming the previous hypothesis. This is similar to the results of a longitudinal study (58) showing that individuals with melancholia are more likely to have a more persistent depressive mood. Previous research (59) has found that the structure and content of individuals’ social networks is partly due to their personality traits. According to the theory of personality traits (60), extroverts tend to be more communicative and sociable, tend to share their experiences with family and friends, and handle emotions better by seeking social support (61). Individuals with low neuroticism tend to view negative events more rationally and calmly (62). Therefore, patients with low neuroticism may view their illness and treatment side effects more calmly. Melancholic temperament belongs to low extraversion and high neuroticism, and this type of patient may be more withdrawn and negative. In addition, according to the theory of temperament (63). Choleric people are excitable and enthusiastic and are good at socializing, but they are often impulsive and irritable mood. Sanguineous people are often warm, lively, compassionate, and sociable, but they can be easily distracted and careless. Lymphatic people are emotionally stable, rational, and thoughtful. Melancholic people are generally withdrawn, less social, more emotional, delicate and sensitive. Melancholis often leads to emotional instability in the face of stress when a pessimistic mood is more likely to appear, and the individual is unable to adjust his or her state of mind in time, resulting in an adverse prognosis (58). Therefore, medical staff should pay more attention to changes in melancholic patients’ mood during hospitalization, help to relieve their negative moods, take reasonable and effective intervention measures, and ensure the intensity and durability of the intervention for the best results.

### Social demography and disease-related factors had no predictive effect on the social avoidance trajectories of patients with breast cancer

Sociodemographic characteristics are often used as important indicators to identify high-risk groups. In the univariate analysis, we found that the average monthly income of the families in Class 1 and Class 2 was lower (*P* < 0.05). As one of the demographic characteristics, economic status is closely related to the generation of negative emotions and worse quality of life (64). For example, studies by Benedict et al. (65) and Fatiregun et al. (66) have shown that breast cancer patients with a financial burden or low income experience greater anxiety. The reasons for this may be that the high cost of treatment, difficulties in employment due to disease factors and changes in employment forms exacerbate the financial problems of breast cancer patients, causing severe financial stress (67, 68). Therefore, some patients may cut back on leisure and partying activities to save money. On the other hand, it may be that low-income people tend to have lower self-esteem than high-income people, which often leads to social anxiety or social stigma due to their unequal status in social activities (69). These direct or indirect effects will have an impact on the patient’s physical and mental health, quality of life and even treatment results and further increase the difficulty of patients re-entering society.

The univariate analysis also found that patients in Class 1 lived in rural areas (*P* < 0.05). Numerous studies (70–72) have shown that patients living in rural areas are more likely to lack self-esteem and be subject to stigma than those living in urban areas, especially female patients (73). This may be because patients living in rural areas are less educated, poorer, and older. Lack of awareness of the disease and negative side effects of the treatment (i.e., breast loss, hair loss, scarring, and other physical effects), in turn, exacerbate the range of psychological distress that breast cancer patients experience, such as fear, low self-esteem, and stigma (74). This is in line with previous studies showing that patients with high shame are more likely to exhibit social avoidance behaviors during social processes, such as avoiding being with others, talking to others, or avoiding contact with others (75).

However, in multivariate logistic regression analysis, after controlling for temperament type, the former two variables did not significantly predict the trajectories of social avoidance. This is consistent with the findings of most longitudinal studies, in which most sociodemographic and disease-related data on patients were not associated with trajectory categories (76). The possible reason is that longitudinal studies are expensive and difficult to conduct compared with cross-sectional studies, so the study sample size is usually small, and further comparative studies are needed. However, healthcare professionals may play a role in minimizing the financial impact of a cancer diagnosis through early assessment, communication of patients’ potential work capacity and appropriate referrals to occupational therapy to aid return to work or financial planning.

## Limitations

A limitation of the study is that it used a convenience sample and consisted of a single-sex population, including only women with breast cancer. These findings may not be able to infer the trajectory of social avoidance in other regions or among survivors of other types of cancer. Second, we excluded patients with psychiatric disorders only through medical records and did not exclude patients with personality disorders through specialized personality disorder diagnostic forms (e.g., SCID), which may have led to bias in our findings. Third, the relatively small sample size, coupled with the accuracy of BIC, may mask the low incidence of undetectable associated categories or may underestimate the relationship between latent category variables and covariates. In addition, the observation time was relatively short compared with the whole disease treatment period, and the changes in social avoidance behavior could not be fully understood during the whole survival period. Finally, loss to follow-up may result in bias in the outcome of observations.

## Conclusion

Our study proved the heterogeneity of social avoidance behavior patterns in Chinese patients with breast cancer and by examining the influencing effects of demographic factors, clinical factors, and temperament types on development of social avoidance behaviors, we identified the subgroup that was at higher risk of developing a serious social avoidance pattern, which is characterized by melancholic temperament. Timely interventions targeting subpopulations should be developed in future studies.

## Data availability statement

The original contributions presented in this study are included in the article/supplementary material, further inquiries can be directed to the corresponding authors.

## Ethics statement

The studies involving human participants were reviewed and approved by KY20192117-F-1. The patients/participants provided their written informed consent to participate in this study. Written informed consent was obtained from the individual(s) for the publication of any potentially identifiable images or data included in this article.

## Author contributions

CH, TY, and CW proposed the experiment, designed the procedure, and performed most of the work on the manuscript. BC and SW made useful comments on the experiment and helped to revise the manuscript several times. YH, YL, JY, SH, and YD helped to find participants and performed the experimental work. All authors contributed to the article and approved the submitted version.

## Abbreviations

GMM: growth mixed model
PTS: posttraumatic stress
PTG: posttraumatic growth
PTSD: posttraumatic stress disorder
LCGM: latent class growth model
AIC: akaike information criterion
BIC: bayesian information criterion
aBIC: sample size adjusted BIC
LRT: likelihood ratio test
BLRT: bootstrapped likelihood ratio test.

## References

[1] Sung H Ferlay J Siegel RL Laversanne M Soerjomataram I Jemal A Global cancer statistics 2020: GLOBOCAN estimates of incidence and mortality worldwide for 36 cancers in 185 countries. *CA Cancer J Clin.* (2021) 71:209–49. 10.3322/caac.21660 33538338

[2] Bodai BI Tuso P. Breast cancer survivorship: a comprehensive review of long-term medical issues and lifestyle recommendations. *Perm J.* (2015) 19:48–79. 10.7812/TPP/14-241 25902343 PMC4403581

[3] Powers N Gullifer J Shaw R. When the treatment stops: a qualitative study of life post breast cancer treatment. *J Health Psychol.* (2014) 21:1371–82. 10.1177/1359105314553963 27357923

[4] Silva AVD Zandonade E Amorim MHC. Anxiety and coping in women with breast cancer in chemotherapy. *Rev Lat Am Enferm.* (2017) 25:e2891. 10.1590/1518-8345.1722.2891 28591299 PMC5479372

[5] Alves PC Santos MCL Fernandes AFC. Stress and coping strategies for women diagnosed with breast cancer: a transversal study. *Online Braz J Nurs.* (2012) 11:305–18. 10.5935/1676-4285.20120028

[6] Fangel L Panobianco M Kebbe L Almeida A Gozzo T. Qualify of life and daily activities performance after breast cancer treatment. *Acta Paul Enferm.* (2013) 26:93–100. 10.1590/S0103-21002013000100015

[7] Xu H Wang H Yuan C Zhai Q Tian X Wu L Identifying diseases that cause psychological trauma and social avoidance by GCN-Xgboost. *BMC Bioinform.* (2020) 21:504. 10.1186/s12859-020-03847-1 33323103 PMC7739481

[8] Miovic M Block S. Psychiatric disorders in advanced cancer. *Cancer.* (2007) 110:1665–76. 10.1002/cncr.22980 17847017

[9] Kaldewaij R Koch SBJ Volman I Toni I Roelofs K. On the control of social approach–avoidance behavior: neural and endocrine mechanisms. *Curr Top Behav Neurosci.* (2016) 30:275–93. 10.1007/7854_2016_446 27356521

[10] Bowker JC Stotsky MT Etkin RG. How BIS/BAS and psycho-behavioral variables distinguish between social withdrawal subtypes during emerging adulthood. *Pers Individ Differ.* (2017) 119:283–8. 10.1016/j.paid.2017.07.043

[11] Ganz PA Bower JE Stanton AL. Special issues in younger women with breast cancer. *Adv Exp Med Biol.* (2015) 862:9–21. 10.1007/978-3-319-16366-6_2 26059926

[12] Jakobsen K Magnus E Lundgren S Reidunsdatter RJ. Everyday life in breast cancer survivors experiencing challenges: a qualitative study. *Scand J Occup Ther.* (2018) 25:298–307. 10.1080/11038128.2017.1335777 28562163

[13] Hinzey A Gaudier-Diaz MM Lustberg MB DeVries AC. Breast cancer and social environment: getting by with a little help from our friends. *Breast Cancer Res.* (2016) 18:54. 10.1186/s13058-016-0700-x 27225892 PMC4881170

[14] Runeson B Rich C. Diagnostic and statistical manual of mental disorders, 3rd ed. (DSM-III), adaptive functioning in young swedish suicides. *Ann Clin Psychiatry.* (1994) 6:181–3. 10.3109/10401239409149001 7881498

[15] Nelson B Yuen HP Wood SJ Lin A Spiliotacopoulos D Bruxner A Long-term follow-up of a group at ultra high risk (“prodromal”) for psychosis. *JAMA Psychiatry.* (2013) 70:793–802. 10.1001/jamapsychiatry.2013.1270 23739772

[16] Carrión RE Correll CU Auther AM Cornblatt BA. A severity-based clinical staging model for the psychosis prodrome: longitudinal findings from the New York recognition and prevention program. *Schizophr Bull.* (2017) 43:64–74. 10.1093/schbul/sbw155 28053131 PMC5216868

[17] Zhao T Hu Y Zang T Cheng L. Identifying alzheimer’s disease-related proteins by LRRGD. *BMC Bioinform.* (2019) 20:570. 10.1186/s12859-019-3124-7 31760934 PMC6876080

[18] Schlund MW Carter H Cudd G Murphy K Ahmed N Dymond S Human social defeat and approach-avoidance: escalating social-evaluative threat and threat of aggression increases social avoidance. *J Exp Anal Behav.* (2021) 115:157–84. 10.1002/jeab.654 33369748 PMC8168404

[19] Kenen R Ardern-Jones A Eeles R. “Social separation” among women under 40 years of age diagnosed with breast cancer and carrying a BRCA1 or BRCA2 mutation. *J Genet Couns.* (2006) 15:149–62. 10.1007/s10897-005-9015-2 16724273

[20] Martino ML Lemmo D Gargiulo A Barberio D Abate V Avino F Underfifty women and breast cancer: narrative markers of meaning-making in traumatic experience. *Front Psychol.* (2019) 10:618. 10.3389/fpsyg.2019.00618 30984067 PMC6448035

[21] Peters J Bellet BW Jones PJ Wu GWY Wang L McNally RJ. Posttraumatic stress or posttraumatic growth? Using network analysis to explore the relationships between coping styles and trauma outcomes. *J Anxiety Disord.* (2021) 78:102359. 10.1016/j.janxdis.2021.102359 33524701

[22] Bonanno GA. Loss, trauma, and human resilience: have we underestimated the human capacity to thrive after extremely aversive events? *Am Psychol.* (2004) 59:20–8. 10.1037/0003-066x.59.1.20 14736317

[23] Tedeschi RG Calhoun LG. The posttraumatic growth inventory: measuring the positive legacy of trauma. *J Trauma Stress.* (1996) 9:455–71. 10.1002/jts.2490090305 8827649

[24] Tedeschi RG Calhoun LG. Posttraumatic growth: conceptual foundations and empirical evidence. *Psychol Inq.* (2004) 15:1–18. 10.1207/s15327965pli1501_01

[25] Parikh D Ieso PD Garvey G Thachil T Ramamoorthi R Penniment M Post-traumatic stress disorder and post-traumatic growth in breast cancer patients – a systematic review. *Asian Pac J Cancer Prev.* (2015) 16:641–6. 10.7314/apjcp.2015.16.2.641 25684500

[26] Bonanno GA Mancini AD. Beyond resilience and PTSD: mapping the heterogeneity of responses to potential trauma. *Psychol Trauma Theory Res Pract Policy.* (2012) 4:74–83. 10.1037/a0017829

[27] Fletcher S O’Donnell M Forbes D. Personality and trajectories of posttraumatic psychopathology: a latent change modelling approach. *J Anxiety Disord.* (2016) 42:1–9. 10.1016/j.janxdis.2016.05.003 27235835

[28] Bergner RM. What is personality? Two myths and a definition. *New Ideas Psychol.* (2020) 57:100759. 10.1016/j.newideapsych.2019.100759

[29] Eysenck HJ. Dimensions of personality: 16, 5 or 3?—criteria for a taxonomic paradigm. *Pers Individ Differ.* (1991) 12:773–90. 10.1016/0191-8869(91)90144-z

[30] Owens GP. Predictors of posttraumatic growth and posttraumatic stress symptom severity in undergraduates reporting potentially traumatic events. *J Clin Psychol.* (2016) 72:1064–76. 10.1002/jclp.22309 27062393

[31] Panjikidze M Beelmann A Martskvishvili K Chitashvili M. Posttraumatic growth, personality factors, and social support among war-experienced young georgians. *Psychol Rep.* (2020) 123:687–709. 10.1177/0033294118823177 30704339

[32] Hassan R Schmidt LA. Trajectories of behavioral avoidance in real time: associations with temperament and physiological dysregulation in preschoolers. *J Exp Child Psychol.* (2021) 209:105177. 10.1016/j.jecp.2021.105177 34089921

[33] Tsai J El-Gabalawy R Sledge WH Southwick SM Pietrzak RH. Post-traumatic growth among veterans in the USA: results from the national health and resilience in veterans study. *Psychol Med.* (2014) 45:165–79. 10.1017/s0033291714001202 25065450

[34] Koutrouli N Anagnostopoulos F Potamianos G. Posttraumatic stress disorder and posttraumatic growth in breast cancer patients: a systematic review. *Women Health.* (2012) 52:503–16. 10.1080/03630242.2012.679337 22747186

[35] Sijbrandij JJ Hoekstra T Almansa J Peeters M Bültmann U Reijneveld SA. Variance constraints strongly influenced model performance in growth mixture modeling: a simulation and empirical study. *BMC Med Res Methodol.* (2020) 20:276. 10.1186/s12874-020-01154-0 33183230 PMC7659099

[36] Infurna FJ Grimm KJ. The use of growth mixture modeling for studying resilience to major life stressors in adulthood and old age: lessons for class size and identification and model selection. *J Gerontol B Psychol Sci Soc Sci.* (2017) 73:148–59. 10.1093/geronb/gbx019 28329850 PMC5927099

[37] Ames ME Wintre MG. Growth mixture modeling of adolescent body mass index development: longitudinal patterns of internalizing symptoms and physical activity. *J Res Adolesc.* (2016) 26:889–901. 10.1111/jora.12239 28453209

[38] Sijbrandij JJ Hoekstra T Almansa J Reijneveld SA Bültmann U. Identification of developmental trajectory classes: comparing three latent class methods using simulated and real data. *Adv Life Course Res.* (2019) 42:100288. 10.1016/j.alcr.2019.04.018 36732968

[39] Twisk J Hoekstra T. Classifying developmental trajectories over time should be done with great caution: a comparison between methods. *J Clin Epidemiol.* (2012) 65:1078–87. 10.1016/j.jclinepi.2012.04.010 22818946

[40] Diallo TMO Morin AJS Lu H. Impact of misspecifications of the latent variance–covariance and residual matrices on the class enumeration accuracy of growth mixture models. *Struct Equ Model Multidiscip J.* (2016) 23:507–31. 10.1080/10705511.2016.1169188

[41] Landa RJ Holman KC Garrett-Mayer E. Social and communication development in toddlers with early and later diagnosis of autism spectrum disorders. *Arch Gen Psychiatry.* (2007) 64:853–64. 10.1001/archpsyc.64.7.853 17606819

[42] Roberts J Crawford H Hogan AL Fairchild A Tonnsen B Brewe A Social avoidance emerges in infancy and persists into adulthood in fragile x syndrome. *J Autism Dev Disord.* (2019) 49:3753–66. 10.1007/s10803-019-04051-8 31165359 PMC6698894

[43] Barcikowski RS Robey RR. Sample size selection in single group repeated measures analysis. *Anal Var.* (1985) 31.

[44] Eysenck HJ Eysenck MW. Personality and individual dierences: a natural science approach. *Pers Individ Differ.* (1985) 9:343–63. 10.1007/978-1-4613-2413-3

[45] Qian MY Wu GC Zhu RC Zhang X. Revised version of eysenck personality questionnaire-chinese version (EPQ-RSC). *J Psychol.* (2000) 32:317–23. 10.1007/s11769-000-0010-0

[46] Watson D Friend R. Measurement of social-evaluative anxiety. *J Consult Clin Psychol.* (1969) 33:448–57. 10.1037/h0027806 5810590

[47] Wang XD Wang XL Ma H. *Rating Scales for Mental Health.* Beijing: Mental Health Magazine Press (1999) p. 161–7.

[48] He CY Guo SJ Lin YW Du YL Gao L Cao BH. Characteristics and predictors of social isolation in female breast cancer patients. *J Nursing.* (2022) 29:6–11. 10.16460/j.issn1008-9969.2022.13.006

[49] Wang MC Bi XY Ye HS. Mixed growth model: analysis of individual development trends in different categories. *Sociol Stud.* (2014) 29:41. 10.19934/j.cnki.shxyj.2014.04.011

[50] Henselmans I Helgeson VS Seltman H de Vries J Sanderman R Ranchor AV. Identification and prediction of distress trajectories in the first year after a breast cancer diagnosis. *Health Psychol.* (2010) 29:160–8. 10.1037/a0017806 20230089

[51] Kant J Czisch A Schott S Siewerdt-Werner D Birkenfeld F Keller M. Identifying and predicting distinct distress trajectories following a breast cancer diagnosis – from treatment into early survival. *J Psychosom Res.* (2018) 115:6–13. 10.1016/j.jpsychores.2018.09.012 30470319

[52] Dorsett DS. The trajectory of cancer recovery. *Sch Inq Nurs Pract.* (1991) 5:175–84. 1763240

[53] Wang X Wang SS Peng RJ Qin T Shi YX Teng XY Interaction of coping styles and psychological stress on anxious and depressive symptoms in Chinese breast cancer patients. *Asian Pac J Cancer Prev.* (2012) 13:1645–9. 10.7314/apjcp.2012.13.4.1645 22799382

[54] Fernández-Theoduloz G Paz V Nicolaisen-Sobesky E Pérez A Buunk AP Cabana Á Social avoidance in depression: a study using a social decision-making task. *J Abnorm Psychol.* (2019) 128:234–44. 10.1037/abn0000415 30920233

[55] Jin R Xie T Zhang L Gong N Zhang J. Stigma and its influencing factors among breast cancer survivors in China: a cross-sectional study. *Eur J Oncol Nurs.* (2021) 52:101972. 10.1016/j.ejon.2021.101972 33991869

[56] Solbraekke KN Lorem G. Breast-cancer-isation explored: social experiences of gynaecological cancer in a norwegian context. *Sociol Health Illn.* (2016) 38:1258–71. 10.1111/1467-9566.12459 27461035

[57] Esplen MJ Wong J Warner E Toner B. Restoring body image after cancer (ReBIC): results of a randomized controlled trial. *J Clin Oncol.* (2018) 36:749–56. 10.1200/jco.2017.74.8244 29356610

[58] Sakai Y Akiyama T Kawamura Y Matsumoto S Tominaga M Kurabayashi L Temperament and melancholic type: path analysis of a prospective study of depressive mood change in a nonclinical population. *Psychopathology.* (2009) 42:249–56. 10.1159/000224148 19521141

[59] Laakasuo M Rotkirch A Berg V Jokela M. The company you keep: personality and friendship characteristics. *Soc Psychol Pers Sci.* (2016) 8:66–73. 10.1177/1948550616662126

[60] Waldherr A Muck PM. Towards an integrative approach to communication styles: the interpersonal circumplex and the five-factor theory of personality as frames of reference. *Communications.* (2011) 36:1–27. 10.1515/comm.2011.001

[61] Afshar H Roohafza HR Keshteli AH Mazaheri M Feizi A Adibi P. The association of personality traits and coping styles according to stress level. *J Res Med Sci.* (2015) 20:353–8. 26109990 PMC4468450

[62] Schmitt N. The interaction of neuroticism and gender and its impact on self-efficacy and performance. *Hum Perform.* (2007) 21:49–61. 10.1080/08959280701522197

[63] Maher BA Maher WB. Personality and psychopathology: a historical perspective. *J Abnorm Psychol.* (1994) 103:72–7. 10.1037/0021-843x.103.1.72 8040484

[64] Dastan NB Buzlu S. Depression and anxiety levels in early stage turkish breast cancer patients and related factors. *Asian Pac J Cancer Prev.* (2011) 12:137–41. 21517246

[65] Benedict C Fisher S Schapira L Chao S Sackeyfio S Sullivan T Greater financial toxicity relates to greater distress and worse quality of life among breast and gynecologic cancer survivors. *Psychooncology.* (2021) 31:9–20. 10.1002/pon.5763 34224603 PMC9809212

[66] Fatiregun OA Olagunju AT Erinfolami AR Fatiregun OA Arogunmati OA Adeyemi JD. Anxiety disorders in breast cancer: prevalence, types, and determinants. *J Psychosoc Oncol.* (2016) 34:432–47. 10.1080/07347332.2016.1196805 27269867

[67] Ngan TT Van Minh H Donnelly M O’Neill C. Financial toxicity due to breast cancer treatment in low- and middle-income countries: evidence from Vietnam. *Supportive Care Cancer.* (2021) 29:6325–33. 10.1007/s00520-021-06210-z 33860362 PMC8464564

[68] Meernik C Sandler DP Peipins LA Hodgson ME Blinder VS Wheeler SB Breast cancer-related employment disruption and financial hardship in the sister study. *JNCI Cancer Spectr.* (2021) 5:kab024. 10.1093/jncics/pkab024 34104865 PMC8178802

[69] Thomas BE Shanmugam P Malaisamy M Ovung S Suresh C Subbaraman R Psycho-socio-economic issues challenging multidrug resistant tuberculosis patients: a systematic review. *PLoS One.* (2016) 11:e0147397. 10.1371/journal.pone.0147397 26807933 PMC4726571

[70] Simmons LA Yang NY Wu Q Bush HM Crofford LJ. Public and personal depression stigma in a rural american female sample. *Arch Psychiatr Nurs.* (2015) 29:407–12. 10.1016/j.apnu.2015.06.015 26577555

[71] Mutiso VN Musyimi CW Nayak SS Musau AM Rebello T Nandoya E Stigma-related mental health knowledge and attitudes among primary health workers and community health volunteers in rural Kenya. *Int J Soc Psychiatry.* (2017) 63:508–17. 10.1177/0020764017716953 28679343

[72] Maulik PK Devarapalli S Kallakuri S Tewari A Chilappagari S Koschorke M Evaluation of an anti-stigma campaign related to common mental disorders in rural India: a mixed methods approach. *Psychol Med.* (2017) 47:565–75. 10.1017/S0033291716002804 27804895 PMC5244444

[73] Schroeder S Tan CM Urlacher B Heitkamp T. The role of rural and urban geography and gender in community stigma around mental illness. *Health Educ Behav.* (2020) 48:63–73. 10.1177/1090198120974963 33218261

[74] Torres E Dixon C Richman AR. Understanding the breast cancer experience of survivors: a qualitative study of African American women in rural Eastern North Carolina. *J Cancer Educ.* (2015) 31:198–206. 10.1007/s13187-015-0833-0 25877467

[75] Suwankhong D Liamputtong P. Breast cancer treatment: experiences of changes and social stigma among thai women in Southern Thailand. *Cancer Nurs.* (2016) 39:213–20. 10.1097/ncc.0000000000000255 25881809

[76] Brandão T Schulz MS Matos PM. Psychological adjustment after breast cancer: a systematic review of longitudinal studies. *Psychooncology.* (2016) 26:917–26. 10.1002/pon.4230 27440317

